# Peroxisomal Dysfunction Contributes to White Matter Injury Following Subarachnoid Hemorrhage in Rats via Thioredoxin-Interacting Protein-Dependent Manner

**DOI:** 10.3389/fcell.2020.576482

**Published:** 2020-10-22

**Authors:** Weilin Xu, Jun Yan, Shuda Chen, Umut Ocak, Anwen Shao, Jianmin Zhang

**Affiliations:** ^1^Department of Neurosurgery, Second Affiliated Hospital, School of Medicine, Zhejiang University, Hangzhou, China; ^2^Department of Neurosurgery, Affiliated Tumor Hospital of Guangxi Medical University, Nanning, China; ^3^Department of Neurosurgical Intensive Care Unit, Second Affiliated Hospital, School of Medicine, Zhejiang University, Hangzhou, China; ^4^Department of Emergency Medicine, Bursa Yuksek Ihtisas Training and Research Hospital, University of Health Sciences, Bursa, Turkey; ^5^Department of Emergency Medicine, Bursa City Hospital, Bursa, Turkey

**Keywords:** subarachnoid hemorrhage, white matter injury, peroxisomes, catalase, thioredoxin-interacting protein

## Abstract

**Background and Purpose:**

White matter injury (WMI) exists in the early stage of subarachnoid hemorrhage (SAH) and has not been well addressed so far.

**Methods:**

We utilized short hairpin RNA (shRNA) and clustered regularly interspaced short palindromic repeats (CRISPR) to verify the role of peroxisomes in WMI following SAH. We evaluated short- and long-term neurobehavior after SAH. Western blotting, immunofluorescence, and Golgi staining techniques were performed to assess the changes in protein levels.

**Results:**

Catalase (CAT) CRISPR treatment significantly attenuated neurological deficits and reduced long-term spatial learning and memory impairments after SAH by increasing the level of myelin basic protein (MBP) while decreasing the levels of amyloid precursor protein (APP), interleukin 6 (IL-6), and tumor necrosis factor (TNF)-α. The use of thioredoxin-interacting protein (TXNIP) shRNA significantly offset the effects of CAT shRNA, and the use of glycerone phosphate acyl transferase (GNPAT) shRNA significantly reversed the effects of CAT CRISPR by decreasing the levels of plasmalogens and reactive oxidative species (ROS).

**Conclusion:**

Peroxisomal dysfunction induced by SAH reversely exacerbated cerebral WMI following SAH, which was at least partly mediated by TXNIP and GNPAT pathways.

## Introduction

Subarachnoid hemorrhage (SAH) is one of the most severe cerebrovascular diseases with a high rate of death and disability. Previous studies have mainly focused on studying the injuries of the cortex or hippocampus following SAH; however, white matter injury (WMI) after SAH has not been well addressed. More than half of the central nervous system is composed of white matter, which is more vulnerable to ischemia/hemorrhagic stroke than gray matter (Pang et al., 2019). In our previous study, we found that WMI occurred in the early stage of SAH, which is characterized by amyloid precursor protein (APP) accumulation, myelin basic protein (MBP) degradation, and white matter edema (Peng et al., 2019). However, the underlying mechanisms of WMI following SAH are still unclear.

Peroxisome is a type of organelle widely found in eukaryotic cells. In the brain, peroxisomes are important for detoxification of reactive oxidative species (ROS) and metabolism of myelin lipids in oligodendrocytes (Kassmann, 2014). Peroxisomal dysfunction in the white matter may result in devastating damages to the myelin, including delayed myelination, hypomyelination, demyelination, and dysmyelination (Trompier et al., 2014). However, the roles of peroxisomes in SAH have not been reported yet. In this study, we hypothesized that peroxisomal dysfunction contributes greatly to WMI following SAH.

## Materials and Methods

The data that support the findings of this study are available from the corresponding author upon reasonable request. All experiments in this study were conducted according to the protocols proposed by the local Institutional Animal Care and Use Committee (IACUC).

We performed endovascular perforation SAH model (Xu et al., 2019) for this study. The degree of SAH was assessed with a new grading system as previously described (Xu et al., 2019). Mortality rate and short-term neurological functions (modified Garcia scoring system and beam balance test) were assessed at 24 h after SAH. Long-term neurologic functions (rotarod test in the 1st, 2nd, and 3rd week and Morris water maze on days 21–25 following SAH) were also evaluated. Western blot analysis, immunofluorescence, and Golgi staining techniques were performed to assess the changes of protein levels. ROS assay was conducted based on the instructions of ROS assay kit (Jiancheng, China).

Data were showed as mean ± standard deviation (SD). If the data met the requirement of satisfied normality and homogeneity of variance, one-way analysis of variance (ANOVA) followed by Tukey’s *post hoc* test for multiple comparisons between different groups was used. For the data that failed the normality test, non-parametric statistics were applied. Additionally, two-way repeated measures ANOVA was applied to analyze the data of long-term neurological functions. Statistical significance was defined as *P* < 0.05. GraphPad Prism (GraphPad Software, San Diego, CA, United States) was applied to analyze the data.

## Results

### Peroxisomal Dysfunction Aggravated Short- and Long-Term Neurobehavioral Dysfunction After SAH

**FIGURE 1:**
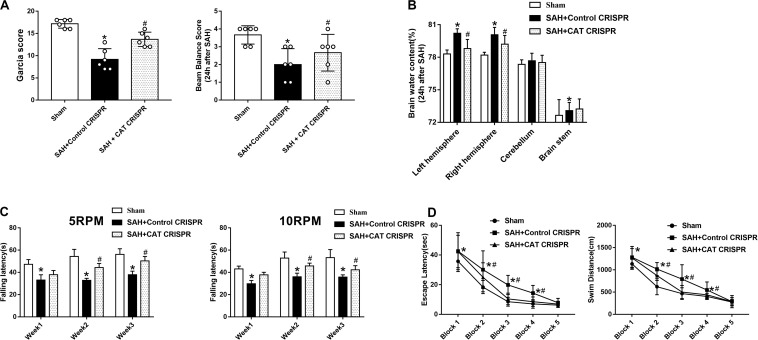
The application of CAT CRISPR greatly attenuated neurological deficits in rats receiving SAH induction. **(A)** Modified Garcia and beam balance tests scores for each group; **(B)** brain water content; **(C)** rotarod test of 5 and 10 RPM; **(D)** escape latency and swim distance of Morris water maze. The bars represent the mean ± SD. *p < 0.05 versus sham, #*p* < 0.05 versus SAH + control CRISPR.

In short-term neurobehavior evaluation, the scores of both modified Garcia and beam balance tests were significantly decreased in the SAH + control clustered regularly interspaced short palindromic repeats (CRISPR) group. However, the use of catalase (CAT) CRISPR significantly improved neurobehavior test scores (*P* < 0.05; Figure 1A). In addition, the use of CAT CRISPR significantly decreased brain water content of the right and left cerebral hemispheres (*P* < 0.05; Figure 1B).

In long-term neurobehavior evaluation, the results revealed that rats in SAH + control CRISPR group had shorter falling latency than the sham group at both 5 and 10 RPMs; However, the rats which received CAT CRISPR treatment stayed for a longer time on the roller in the 1st, 2nd, and 3rd week after SAH (*P* < 0.05; Figure 1C).

In Morris water maze, there was no significant difference of velocity between different groups (Supplementary Figure 3). However, the rats that underwent SAH induction showed a significant decrease in escape latency in blocks 1, 2, 3, and 4 and also a longer distance to find the platform in blocks 1, 2, 3, and 4 than the rats in the sham group. However, these situations were significantly improved by CAT CRISPR treatment (*P* < 0.05; Figure 1D). For probe trials, the results showed that the rats subjected to SAH stayed for a shorter time (16%) in the target quadrant compared to the sham group (38%). The CAT CRISPR treatment significantly reversed the results (26%, Supplementary Figure 4).

### Promotion of Peroxisomal Functions With CAT CRISPR Provided Neuroprotective Effects After SAH

**FIGURE 2:**
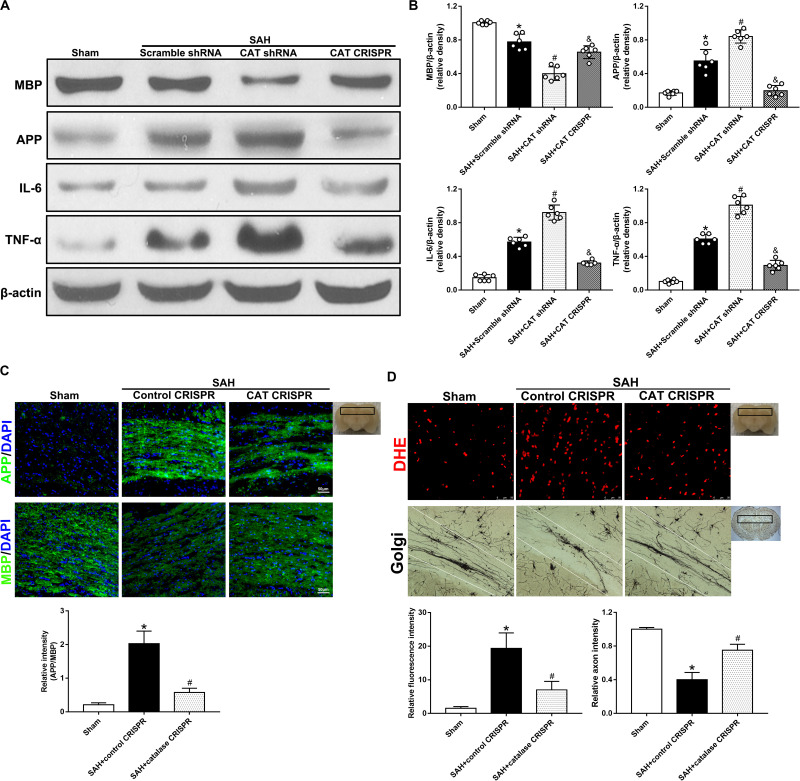
The application of CAT CRISPR attenuated WMI by decreasing the levels of inflammatory factors and ROS. **(A,B)** Representative western blot images and quantitative analyses of MBP, APP, IL-6, and TNF-α; **(C)** immunofluorescence staining and quantitative analyses of APP and MBP; **(D)** representative pictures and quantitative analysis of DHE staining and Golgi staining. The bars represent the mean ± SD. *p < 0.05 versus sham, #*p* < 0.05 versus SAH + control CRISPR, &*p* < 0.05 versus SAH + CAT shRNA.

**FIGURE 3:**
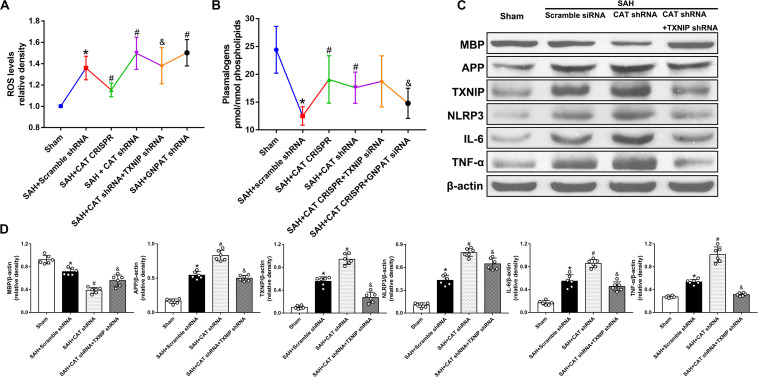
Peroxisomal dysfunction exacerbates cerebral WMI following SAH via TXNIP and GNPAT pathways. **(A)** Level of ROS, &*p* < 0.05 versus SAH + CAT shRNA; **(B)** level of plasmalogen, &*p* < 0.05 versus SAH + CAT CRISPR; **(C,D)** representative western blot image and quantitative analyses of MBP, APP, TXNIP, NLRP3, IL-6, and TNF-α. The bars represent the mean ± SD. *p < 0.05 versus sham, #*p* < 0.05 versus SAH + scramble shRNA, &*p* < 0.05 versus SAH + CAT shRNA.

Western blot analysis revealed that the induction of SAH significantly decreased the protein expression level of MBP but increased the levels of APP, interleukin 6 (IL-6), and tumor necrosis factor (TNF)-α (*P* < 0.05; Figures 2A,B). Besides, immunofluorescence staining showed that the relative intensity ratio of APP/MBP was significantly increased in the SAH + control CRISPR group; however, it was significantly decreased in the SAH + CAT CRISPR group (*P* < 0.05; Figure 2C). CAT CRISPR treatment decreased the levels of ROS and dihydroethidium (DHE)-positive cells (*P* < 0.05; Figures 2D, 3A) as well. In Golgi staining, the induction of SAH significantly increased the number of broken axons, which were greatly reversed by CAT CRISPR treatment (*P* < 0.05; Figure 2D).

### Thioredoxin-Interacting Protein/NOD-Like Receptor Protein Pathway Contributed to the WMI Following SAH Due to Peroxisomal Dysfunction

Catalase short hairpin RNA (shRNA) and thioredoxin-interacting protein (TXNIP) shRNA were administered to verify the role of TXNIP in peroxisomal dysfunction-mediated WMI following SAH. The use of CAT shRNA significantly decreased the protein level of MBP but increased that of APP, TXNIP, NOD-like receptor protein (NLRP3), IL-6, and TNF-α (*P* < 0.05; Figures 3C,D). On the other hand, the level of ROS was increased in the CAT shRNA group while plasmalogens was decreased. However, these results were reversed by the application of TXNIP shRNA except for the level of plasmalogens (*P* < 0.05; Figures 3A,B).

### Glycerone Phosphate Acyl Transferase Was Involved in Peroxisomal Dysfunction Mediated WMI Following SAH

In order to further explore the role of plasmalogens in peroxisomal dysfunction-mediated WMI, we administered glycerone phosphate acyl transferase (GNPAT) shRNA. The results showed that the levels of plasmalogens were significantly increased in the SAH + CAT CRISPR group whereas they were decreased after the use of GNPAT shRNA (*P* < 0.05; Figure 3B). Besides, the level of ROS was significantly increased after the use of GNPAT shRNA when compared with the SAH + Scramble shRNA group (*P* < 0.05; Figure 3A).

### The Efficacy of CAT CRISPR and shRNA in Promotion or Knockdown of Catalase

We have used naive animals to test the efficacy of CAT CRISPR and shRNA. The results showed that the administration of CAT CRISPR significantly promoted the expression of catalase by 44%, and the administration of shRNA significantly decreased the expression of catalase by 56% (Supplementary Figure 5).

## Discussion

A catalase is one of the crucial antioxidant enzymes that plays an important role in protecting the cell from oxidative damage by catalyzing the decomposition of hydrogen peroxide to water and oxygen (Chelikani et al., 2004). Recent studies have shown that the level of catalase significantly increases after SAH, probably due to the induction by H_2_O_2_ (Kuo et al., 2013). Moreover, catalase mainly resides in the peroxisome, accounting for 30% of all peroxisomal enzymes. Therefore, in this study, we elected to proceed with catalase as the marker to represent the functional status of peroxisomes.

In order to evaluate the role of dysfunctional peroxisomes in SAH, we administered CAT shRNA and CAT CRISPR to knockdown and promote the expression of catalase, respectively, which is the main functional protein and marker of peroxisomes. Our results showed that rats in the SAH group showed decreased motor functions and delayed responses that were significantly improved by the promotion of catalase expression with CRISPR. In addition, in long-term neurological function evaluation, rats subjected to SAH showed notably decreased cognitive and memory functions when compared with those that underwent catalase promotion.

Motor deficits and cognitive dysfunctions have been observed in association with WMI in patients with SAH (Yeo et al., 2012). In the past few years, many researchers have tried to unveil the potential mechanisms underlying SAH. Kummer et al. (2015) and Wu et al. (2017) have both indicated that a mechanical pressure contributes to SAH-associated WMI. Wu et al. (2017) have also showed that WMI after SAH may result due to glial response and white matter ischemia. In an experimental SAH model, Egashira et al. (2015) have demonstrated that increased secretion of MMP-9 from astrocytes and oligodendrocyte precursors caused disruption of the blood–brain barrier (BBB) and subsequent WMI following SAH. Pang et al. (2019) have summarized the pathogenesis and underlying mechanisms of WMI after SAH in their systematic review. In this review, authors have indicated that WMI mainly results from five pathophysiologic changes, including BBB disruption, physical and mechanical damage, neuroinflammation, ischemia, and oxidative stress, underlining the importance of targeting WMI in the treatment of SAH.

It should be noted that some recent studies have reported the importance of peroxisomes in the maintenance of white matter integrity (Hulshagen et al., 2008). Under lights of the literature evidence summarized above, we explored the roles of peroxisomes in WMI following SAH. First, in the results of western blot and immunofluorescence staining, we have found that the level of white matter marker, MBP, was decreased while the marker for WMI, APP, was increased after SAH. Similarly, in the Golgi staining, we saw that the fibers were seriously damaged in the SAH group. However, these changes were reversed by the administration of catalase CRISPR. Moreover, as peroxisomes have important functions in the metabolism of lipids and the balance of redox and neuroinflammation, we also evaluated the changes of lipids, ROS, and inflammatory factors. It has been reported that the formation of plasmalogen starts in peroxisomes and plasmalogen is the most abundant phospholipid in myelin (Kassmann, 2014). Deficiency of plasmalogens causes profound abnormalities in the myelination of nerve cells (Hulshagen et al., 2008). The results in the present study revealed that peroxisomal dysfunction significantly increased the levels of ROS and inflammatory factors (IL-6 and TNF-α) but decreased the level of plasmalogen, which can be significantly abolished by the use of catalase CRISPR.

We have also administered TXNIP shRNA to explore the underlying mechanism of WMI caused by peroxisomal dysfunction following SAH. TXNIP is an endogenous inhibitor of the thioredoxin (TRX) system, a major cellular thiol-reducing and antioxidant system (Xu et al., 2019). Recently, a significant body of literature has supported an essential role of TXNIP in the activation of the NLRP3-inflammasome and its downstream molecules (Nasoohi et al., 2018). Various endogenous and exogenous stimuli control TXNIP expression, including glucose, ER stress, oxidative stress, and AMPK. Noteworthy, ROS-generating stimuli may potentiate the TXNIP/NLRP3 pathway by enhancing TXNIP transcription and expression. The underlying mechanisms of ROS-mediated TXNIP elevation may be dependent on MAPK, Forkhead box class O (FOXO), etc., (Nasoohi et al., 2018). Additionally, the accumulation of H_2_O_2_ greatly increased the level of TXNIP (Zaragoza-Campillo and Moran, 2017), suggesting the potential association of TXNIP and peroxisome-mediated ROS. In our study, the levels of TXNIP, ROS, NLRP3, inflammatory factors, and APP were significantly increased after SAH. However, these effects were abolished by the administration of TXNIP shRNA. The results above have suggested that WMI caused by peroxisomal dysfunction were transmitted via the TXNIP-mediated pathway. Interestingly, the level of plasmalogen was not influenced by the administration of TXNIP shRNA. After carefully reading the literatures and being proved with experiments, we have found that lipids may change by peroxisomal dysfunction and subsequent WMI may be dependent on the functional status of GNPAT, the key enzyme for the initial steps of plasmalogen biosynthesis. Consequently, genetic mutations in the GNPAT gene can result in plasmalogen deficiencies, leading to great injuries in the white matter (Malheiro et al., 2019). Besides, plasmalogens have been demonstrated to protect mammalian cells against the damaging effects of ROS (Hulshagen et al., 2008). In order to prove this, we have applied GNPAT shRNA. The results showed that GNPAT shRNA greatly decreases the levels of plasmalogen and reduces the effects of catalase CRISPR. Besides, after the use of GNPAT shRNA, the level of ROS was decreased as well, further demonstrating that peroxisomal dysfunction-induced plasmalogen and ROS both contribute to WMI following SAH.

## Conclusion

These data provide unprecedented evidence that peroxisomal dysfunction induced by SAH reversely exacerbates cerebral WMI following SAH, which was possibly mediated by TXNIP and GNPAT pathways.

## Data Availability Statement

The raw data supporting the conclusions of this article will be made available by the authors, without undue reservation.

## Ethics Statement

The animal study was reviewed and approved by the Institutional Animal Care and Use Committee of Zhejiang University.

## Author Contributions

WX and JY designed the study. WX, JY, and AS completed the experiments. WX and AS performed the statistical analysis. WX and JY finished writing the manuscript. SC and UO participated in the revision. JZ and WX participated in discussion development and provided expert guidance. All authors contributed to the article and approved the submitted version.

## Conflict of Interest

The authors declare that the research was conducted in the absence of any commercial or financial relationships that could be construed as a potential conflict of interest.
