# Supplementary material for: Peroxisomal Dysfunction Contributes to White Matter Injury Following Subarachnoid Hemorrhage in Rats via Thioredoxin-Interacting Protein-Dependent Manner
Source: Front Cell Dev Biol. 2020 Oct 22;8:576482. doi: 10.3389/fcell.2020.576482 (PMC7642982; doi:10.3389/fcell.2020.576482)
Supplement: Supplementary file 1 [file Data_Sheet_1.docx]

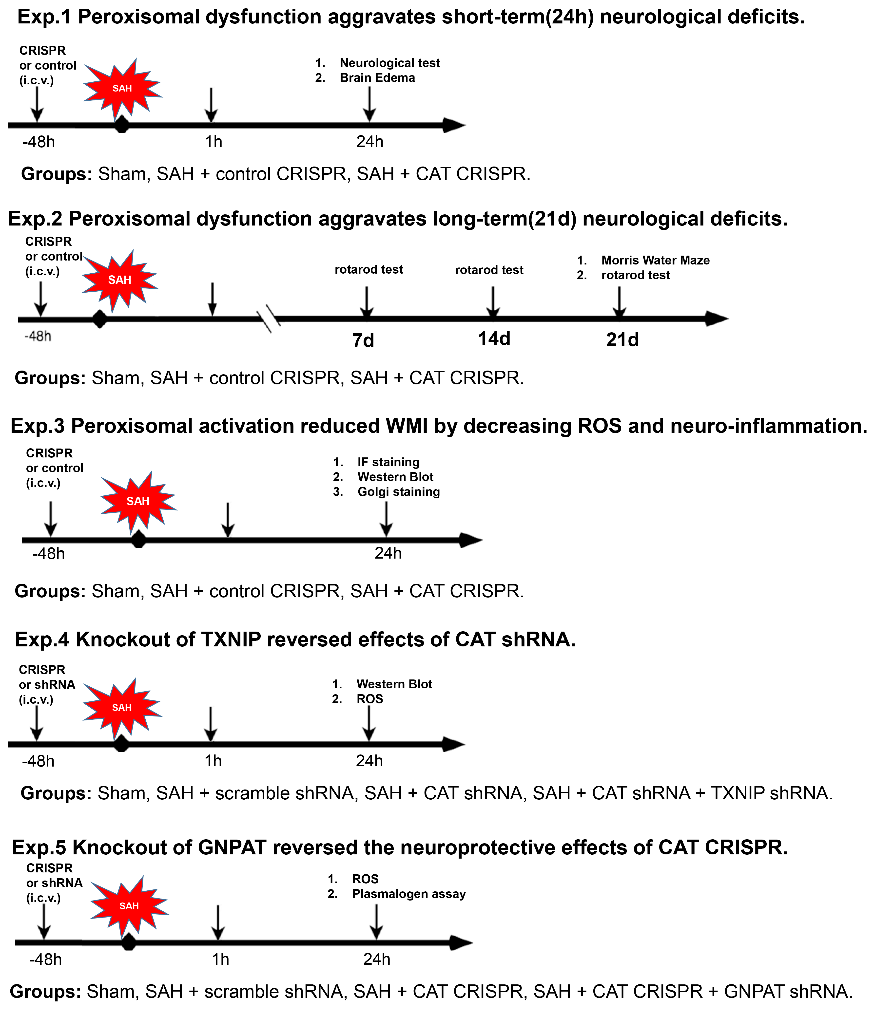


**Supplemental Figure Ⅰ.** Experimental design and animal groups.


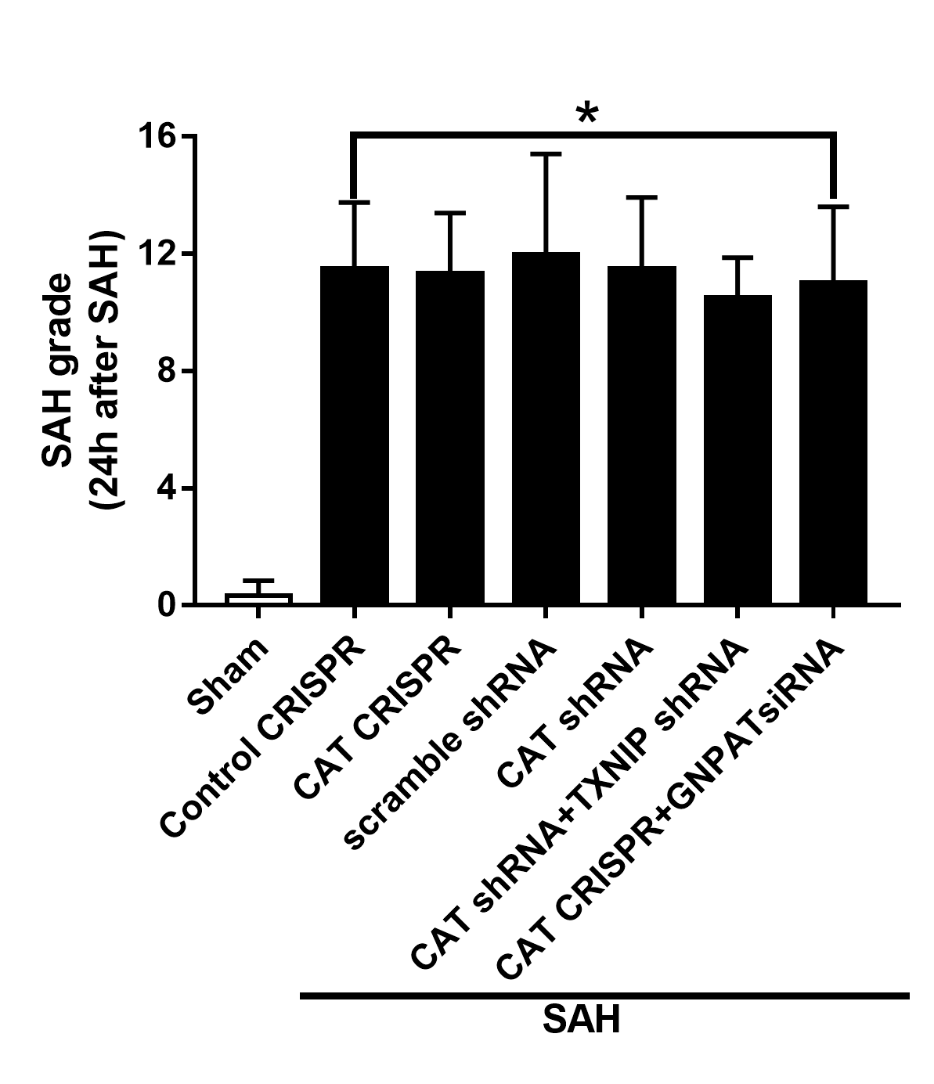


**Supplemental Figure Ⅱ.** SAH grade of each group. The bars represent the mean ± SD. *p<0.05 versus sham, #p<0.05 versus SAH + vehicle at 24 h. n = ?????


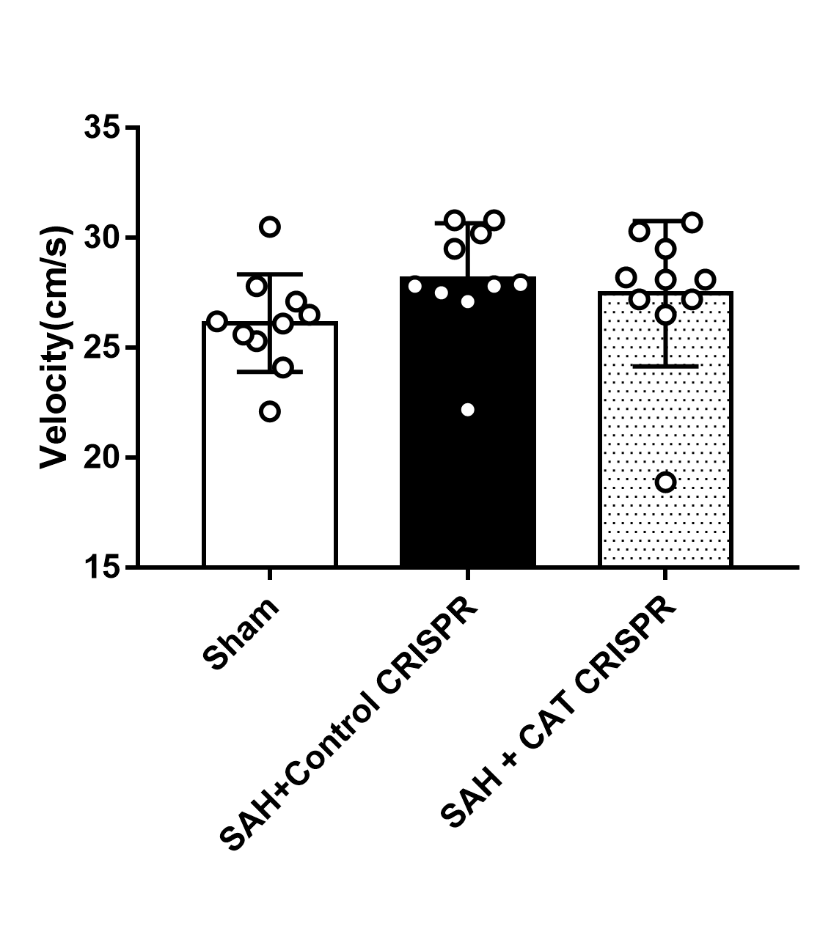


**Supplemental Figure Ⅲ.** The swim speed of rats during Morris water maze test. The bars represent the mean ± SD. n = 10 per group.


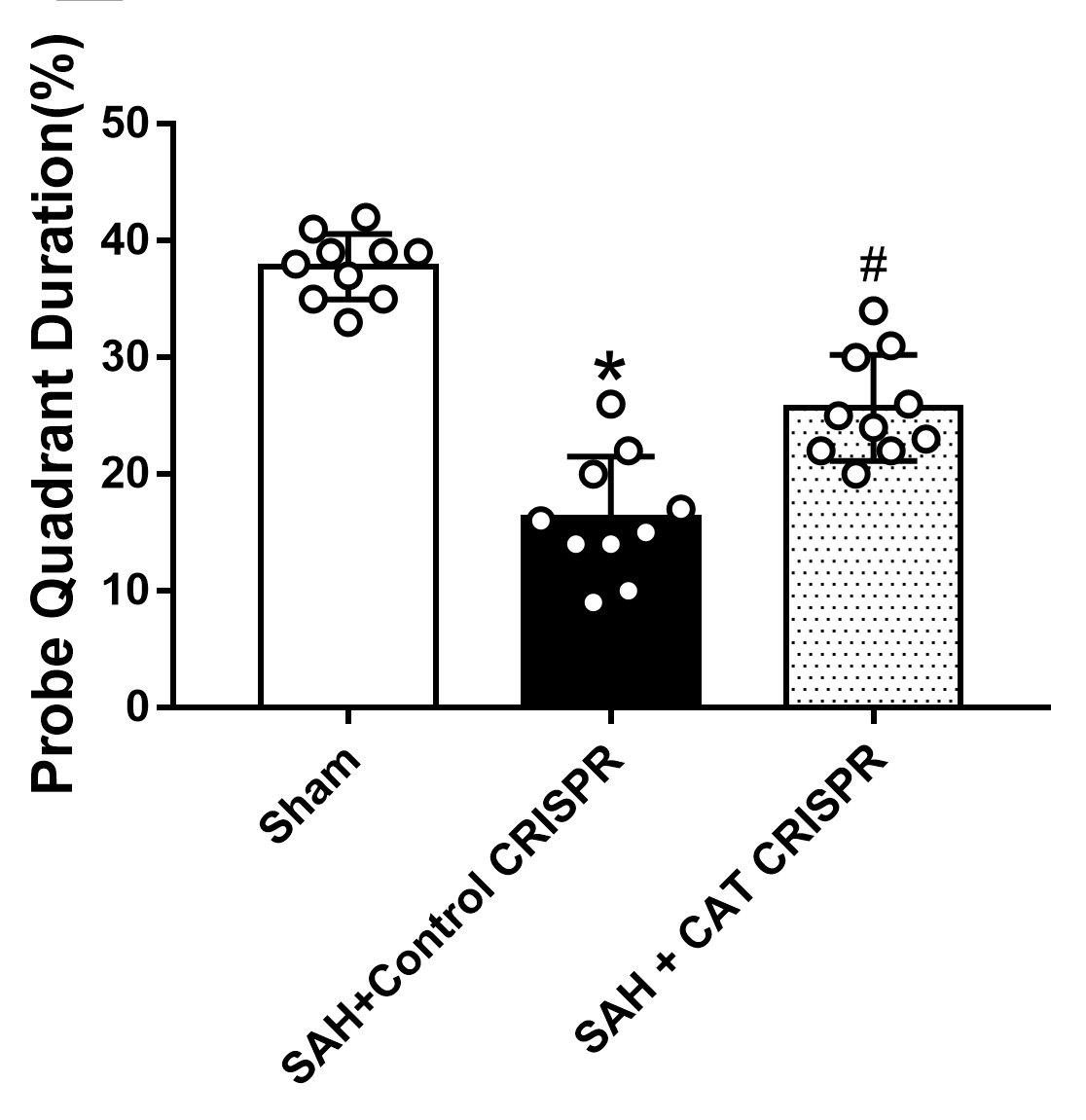


**Supplemental Figure Ⅳ.** Probe quadrant duration of Morris water maze test. The bars represent the mean ± SD. *p<0.05 versus sham, #p<0.05 versus SAH + control CRISPR. n = 10 per group.





**Supplemental Figure Ⅴ.** The efficacy of CRISPR or shRNA in promotion or knockdown of catalase, respectively. Representative Western blot image and quantitative analyses of catalase. The bars represent the mean ± SD. *p<0.05 versus naive. n = ?????

| **Supplemental table Ⅰ.** Neurological Evaluation: Modified Garcia score. | | | | |
| --- | --- | --- | --- | --- |
| **Test** | **Score** | | | |
|  | **0** | **1** | **2** | **3** |
| Spontaneous Activity (in cage for 5 min) | No movement | Barely moves position | Moves but does not approach at least three sides of cage | Moves and approaches at least three sides of cage |
| Spontaneous movements of all limbs | No movement | Slight movement of limbs | Moves all limbs but slowly | Move all limbs same as pre-SAH |
| Movements of forelimbs (outstretching while held by tail) | No outreaching | Slight outreaching | Outreach is limited and less than pre-SAH | Outreach same as pre-SAH |
| Climbing wall of wire cage |  | Fails to climb | Climbs weakly | Normal climbing |
| Reaction to touch on both side of trunk |  | No response | Weak response | Normal response |
| Response to vibrissae touch |  | No response | Weak response | Normal response |

| **Supplemental table Ⅱ.** Neurological Evaluation: Beam balance test | | | | | |
| --- | --- | --- | --- | --- | --- |
|  | 0 | 1 | 2 | 3 | 4 |
| Beam walking (60 sec) | No walking and falls off | No walking but remains on beam | Walking but falls off | Walking less than 20 cm | Walking beyond 20 cm |

**Supplemental Table Ⅲ.** Plans of Morris water maze test.

|  | **Day 1** | | **Day 2** | **Day 3** | **Day 4** | **Day 5** | **Day 6** |
| --- | --- | --- | --- | --- | --- | --- | --- |
|  | **Platform location** | **Starting direction** | **Platform Location: SW Starting Location as follows** | | | | **No platform** |
| **Trial 1** | SW | S | W | N | N | E | N |
| **Trial 2** | NW | N | S | W | E | S |  |
| **Trial 3** | NE | E | N | E | W | W |  |
| **Trial 4** | SE | W | E | S | S | N |  |

S, south; N, north; E, east; W, west.

| **Supplemental Table Ⅳ. Rats physiological data after surgeries (dead and excluded animals are not included).** | | | | | | | |
| --- | --- | --- | --- | --- | --- | --- | --- |
| Group | Rats used | T (℃) | HR (/min) | BP (mmHg) | BG (mmol/L) | PO2 (mmHg) | PCO2 (mmHg) |
| Sham | 42 | 37.6±0.19 | 368±5.1 | 133±0.72 | 6.5±0.29 | 74±2.1 | 39±1.8 |
| SAH + Control CRISPR | 30 | 37.5±0.23 | 362±7.6 | 130±0.87 | 6.4±0.38 | 73±2.8 | 40±2.0 |
| SAH + CAT CRISPR | 42 | 37.7±0.18 | 359±7.3 | 131±0.96 | 6.3±0.29 | 72±2.9 | 39±2.7 |
| SAH + scramble shRNA | 18 | 38.0±0.22 | 364±5.8 | 132±1.21 | 6.7±0.48 | 73±2.7 | 38±2.5 |
| SAH + CAT shRNA | 12 | 37.5±0.19 | 357±6.2 | 129±0.79 | 6.6±0.37 | 74±3.1 | 39±3.8 |
| SAH + CAT shRNA + TXNIP shRNA | 12 | 37.7±0.18 | 361±5.6 | 134±1.31 | 6.4±0.51 | 73±2.9 | 40±2.6 |
| SAH + CAT CRISPR + GNPAT shRNA | 12 | 37.8±0.21 | 362±6.9 | 129±0.84 | 6.5±0.37 | 75±3.3 | 38±2.1 |

**Supplemental Table Ⅴ. Study design and distribution of the animal used.**

| **Groups** | **Mortality** | **Excluded** | **Shared** |
| --- | --- | --- | --- |
| **Exp. 1** |  |  |  |
| Sham | 0% (0/6) | 0 | 0 |
| SAH + Control CRISPR | 14.3% (1/7) | 0 | 0 |
| SAH + CAT CRISPR | 0% (0/6) | 1 |  |
| **Exp. 2** |  | 0 |  |
| Sham | 0% (0/10) | 0 | 0 |
| SAH + Control CRISPR | 16.7% (2/12) | 0 | 0 |
| SAH + CAT CRISPR | 16.7% (2/12) | 0 | 0 |
| **Exp. 3** |  | 0 |  |
| Sham | 0% (0/14) | 0 | 0 |
| SAH + Control CRISPR | 12.5% (2/16) | 1 | 0 |
| SAH + CAT CRISPR | 6.7% (1/15) | 1 | 0 |
| **Exp. 4** |  | 0 |  |
| Sham | 0% (0/6) | 0 | 6 |
| SAH + scramble shRNA | 7.7% (1/13) | 0 | 0 |
| SAH + CAT shRNA | 14.3% (2/14) | 1 | 0 |
| SAH + CAT shRNA + TXNIP shRNA | 14.3% (2/14) | 0 | 0 |
| **Exp. 5** |  | 0 |  |
| Sham | 0% (0/6) | 0 | 6 |
| SAH + scramble shRNA | 0% (0/6) | 0 | 6 |
| SAH + CAT CRISPR | 7.7% (1/13) | 0 | 0 |
| SAH + CAT CRISPR + GNPAT shRNA | 14.3% (2/14) | 1 | 0 |
| **Total** |  |  |  |
| **Sham** | 0% (0/42) |  |  |
| **SAH** | 11.3% (16/142) | 5 | 18 |

**Supplemental Methods and materials**

**Animals**

All animal experiments were performed according to the Institutional Animal Care and Use Committee of Zhejiang University. The procedures were conducted according to the National Institutes of Health’s Guide for the Care and the Use of Laboratory Animals and the ARRIVE (Animal Research: Reporting In Vivo Experiments) guidelines. In detail, we used 280-330g male Sprague–Dawley rats (SLAC Laboratory Animal Co., Ltd. Shanghai, China) for this study. All rats were kept in constant temperature (22 ± 1°C) and humidity (60 ± 5%) on a 12 h day/night cycle, with food and water provided *ad libitum*. The whole study was composed of five different experiments (supplemental Fig. Ⅰ).

**Experimental design**

The whole study was composed of five different experiments, which are shown in supplemental Fig. Ⅰ.

**Experiment 1**. The CAT CRISPR was used to evaluate the role of peroxisomes in short-term (24 h) outcomes. Rats were randomly distributed into three groups: Sham, SAH + control CRISPR (4μg, i.c.v.) and SAH + CAT CRISPR (4μg, i.c.v.). CRISPR was intracerebroventricularly given 48 h before SAH. The rats in sham group received the same procedures as SAH + control CRISPR group except injection. Neurological score and brain water content were measured 24 h after SAH in all groups.

**Experiment 2.** The CAT CRISPR was used to evaluate the role of peroxisomes in long-term (28 days) outcomes. Rats were randomly distributed into three groups: Sham, SAH + control CRISPR (4μg, i.c.v.) and SAH + CAT CRISPR (4μg, i.c.v.). CRISPR was intracerebroventricularly given 48 h before SAH. The rats in the sham group underwent the same procedures as SAH + control CRISPR group except injection. Rotarod test was used to assess long-term neurobehavior in the 1^st^, 2^nd^, and 3^rd^ week and Morris water maze test to evaluate the spatial learning and memory abilities on days 21 to 25 following SAH.

**Experiment 3.** The CAT CRISPR was used to evaluate the effects of dysfunctional peroxisomes of WMI and inflammatory factors 24 h after SAH. Rats were randomly distributed into three groups: Sham, SAH + control CRISPR (4μg, i.c.v.) and SAH + CAT CRISPR (4μg, i.c.v.). CRISPR was intracerebroventricularly given 48 h before SAH. The rats in sham group underwent the same procedures as SAH + control CRISPR group except injection. Western blotting, immunofluorescence staining, as well as Golgi staining were conducted 24 h after SAH.

**Experiment 4** In order to study the role of TXNIP in peroxisomal dysfunction mediated WMI following SAH, TXNIP shRNA was administered in this experiment. shRNA was injected intracerebroventricularly at 48 h before the induction of SAH. Rats were randomly distributed into four groups: Sham, SAH + scramble shRNA (500 pmol, i.c.v.), SAH + CAT shRNA (500 pmol, i.c.v.), and SAH + CAT shRNA + TXNIP shRNA (500 pmol, i.c.v.). The rats in the sham group underwent the same procedures as the SAH + scramble shRNA group except the injection. The ipsilateral/left cerebral cortex from each group was sampled for western blot analysis, plasmalogens and ROS assay at 24 h after SAH.

**Experiment 5** In order to study the role of GNPAT in peroxisomal dysfunction mediated WMI following SAH, GNPAT shRNA was applied in this experiment. shRNA was injected intracerebroventricularly at 48 h before the induction of SAH. The rats were randomly distributed into five groups: Sham, SAH + scramble shRNA (500 pmol, i.c.v.), SAH + CAT shRNA (500 pmol, i.c.v.), SAH + CAT CRISPR (4μg, i.c.v.) and SAH + CAT shRNA + GNPAT shRNA (500 pmol, i.c.v.). The rats in the sham group underwent the same procedures as the SAH + scramble shRNA group except injection. The ipsilateral/left cerebral cortex from each group was sampled for plasmalogens and ROS assay at 24 h after SAH.

**SAH animal model**

We performed a widely used and well-recognized endovascular perforation SAH model [1] for this study. The rats that received anesthesia with 5% isoflurane were intubated and then positioned supine on a ventilator. The rats were kept under maintenance anesthesia with 3% isoflurane with 65/35% medical air/oxygen during the surgery. Rats were closely monitored for heart and respiration rates throughout the procedure. First, we exposed the carotid artery and its bifurcation. Afterwards, a 4-0 sharpened nylon suture was inserted from the external carotid artery. The suture then went along the internal carotid artery and finally reached the bifurcation of the anterior and middle cerebral arteries, where a perforation was executed. The degree of SAH was assessed with a new grading system as previously described [1].

**Drug administration and intracerebroventricular injection**

The rat shRNA (OriGene Technologies, Inc.) and CRISPR (Applied Biological Materials Inc.) were injected into the right ventricle (3.5 mm depth below the skull) 48 h before induction of SAH. The rats were anesthetized with 5% isoflurane, and remained under anesthesia as described above. Next, we used a drill to make a burr hole 1 mm posterior to the bregma and 1.5 mm right lateral to the midline, at which point the drug was slowly administered (3.5 mm in depth, 0.5 μl/min). The needle was kept in place for 5 minutes, and then slowly withdrawn over a period of 5 minutes. Finally, the burr hole and incision were closed with bone wax and sutures, respectively. All surgical procedures were conducted with sterile techniques.

**Mortality, SAH grade and short-term neurological assessment**

Mortality rate and neurological functions (modified Garcia scoring System [1] and beam balance test [1]) were conducted by an independent researcher at 24 h after SAH. Detailed information of neurobehavior evaluation was summarized in supplemental table Ⅰ and Ⅱ.

**Long-term neurological evaluation**

We performed rotarod test to assess long-term neurologic functions in the 1^st^, 2^nd^, and 3^rd^ weeks and Morris water maze test to evaluate the spatial learning and memory abilities on days 21 to 25 following SAH, the procedures of which were previously reported [1].

The motor-sensory deficits were assessed using the rotarod test as previously described [1]. Briefly, rotarod test consists of a rotating horizontal cylinder (7 cm diameter) that is divided into 9.5-cm-wide lanes. Animals had to keep walking forward after being placed on the cylinder. The cylinder started at 5 revolutions per minute (RPM) and 10 RPM, respectively, and accelerated by 2 RPM every 5 s. Latency to fall off was recorded by a photobeam circuit.

The Morris water maze assessed the spatial learning memory and cognitive function as previously described [1]. Briefly, a platform was placed at the center of one of the quadrants. The rats were placed using a semi-random set of start locations to find a visible platform above the water level in 60 s. After that, the rats were guided to the platform and stayed there for 5 s. The probe trial was performed on the last day. During this test, the rats were allowed to swim to search the platform submerged in the water. Swim path, swim distance, escape latency, and probe quadrant duration were recorded by a computerized tracking system. The detailed plans for Morris water maze is summarized in supplemental table Ⅲ.

**Western blot analysis.**

We performed the western blotting as previously described [1]. Under deep anesthesia, rats received trans-cardiac perfusion (0.1 M PBS). The ipsilateral/left cerebral cortex was then sampled for western blotting. In brief, 40 μg of protein from each sample underwent electrophoresis, and then the protein was transferred onto nitrocellulose membranes (100 V, 80 minutes). Afterwards, the membranes were incubated with the following primary antibodies overnight at (4°C): APP (1:1500, Abcam ab32136), MBP (1:1000, Abcam ab40390), TXNIP (1:2000, Abcam ab188865), NLRP3 (1:1000, NOVUS, CO), MBP (1:1000, Abcam ab9324), IL-6 (1:2000, Abcam ab9324), TNF-α (1:5000, Abcam ab6671), and β-actin (1: 3000, Santa Cruz sc-47778). Secondary antibodies were then applied at room temperature for 1 h. Finally, the bands were probed with ECL Plus chemiluminescence reagent Kit (Amersham Biosciences, Arlington Heights, PA), and visualized using an imaging system (Bio-Rad, Versa Doc, model 4000). Next, Image J software (NIH) was used to measure intensity. The results were displayed as relative density (grayscale value of the target proteins /β-actin or total proteins). During western blot quantification, when proteins were detected, the beta-actin of the same sample derived from the same membrane was also detected simultaneously, normalizing all proteins with their own beta-actin.

**Immunofluorescence staining**

After being anesthetized with 5% isoflurane, the rats received trans-cardiac perfusion with 0.1M PBS, followed by 10% formalin. Afterwards, the brains were collected quickly and placed in 10% formalin (4°C, 24 h). The formalin was replaced with 30% sucrose solution for 3 days. The brains were cut into 10 μm sections. The slices were then fixed on slides and used for immunofluorescence staining. The brain slices were incubated at 4°C overnight with the following primary antibodies: APP (1:200, Abcam ab32136), MBP (1:200, Abcam ab40390). Secondary antibodies were then applied at room temperature (21 ℃) for 2 h and the samples were assessed with a fluorescence microscope (Leica Microsystems, Germany). Dihydroethidium (DHE) staining was conducted to assess the level of ROS using the DHE staining kit (DHE, Thermo Fisher Scientific, USA) according to the manufacturer's instructions. DHE positive neurons were counted in the ipsilateral/left cerebral cortex by an independent researcher.

**Golgi staining**

We performed Golgi staining according as previously described [2]. Briefly, the rat brain tissues were removed quickly under deep anesthesia on day 28 after SAH. The freshly dissected brains were immersed in solution A and B for 2 weeks at room temperature and transferred into solution C for 72 h at 4 °C. The brains were sliced using a cryostat system (CM3050S; Leica Microsystems, Bannockburn, III, Germany) at a thickness of 100 μm. The following staining steps were completed according to the FD Rapid GolgiStain™ Kit manufacturer's protocol (Columbia, USA). Brightfield images for brain samples were obtained using an Olympus BX51 microscope (Olympus, Waltham, USA). Microphotographs were analyzed using Image Pro Plus 6.0 software (Media-Cybernetics, Bethesda, MD, USA).

**ROS assay**

We performed ROS assay based on instructions of ROS assay kit (JianCheng, China). Detailed procedures were reported in our recent study [3].

**Statistical analysis**

We organized and displayed the data by mean ± standard deviation (SD). First, we conducted power analysis based on α = 0.05 and the number of groups by the SPSS 22.0 software (IBM, USA), with a power of 0.85 or higher indicating reliable evidence. Then, normality of the data was assessed. If the data met the requirement of satisfied normality and homogeneity of variance, one-way analysis of variance (ANOVA) followed by Tukey’s post hoc test for multiple comparisons between different groups was used. For the data that failed the normality test, non-parametric statistics were applied. Additionally, two-way repeated measures ANOVA was applied to analyze the data of long-term neurological functions. Statistical significance was defined as *P* < 0.05. GraphPad Prism (GraphPad Software, San Diego, CA, USA) was applied to analyze the data.

**Supplemental results**

**Physiological data**

Physiological parameters, including body temperature, blood pressure, heart rate, blood glucose, PO_2_, and PCO_2_ were recorded during surgery. No significant differences were observed between groups (Supplemental Table Ⅳ).

**Mortality Rates and SAH Grade Score**

Totally, 168 rats were used in the present study not including dead and excluded animals. Among these, 42 were in the sham group whereas 126 rats were subjected to SAH. Sixteen rats died and 5 rats were excluded as their SAH grades were less than 7 (Supplemental Table Ⅴ). We then added new animals to replace the excluded rats in order to ensure that there were six rats in each group eventually. Eighteen rats were shared among different groups according to the “Three Rs principle (replacement, reduction and refinement)” [4]. Blood clots were mainly observed around the circle of Willis and ventral brain stem after the induction of SAH. We elected to proceed with basal cortex region for this study. There was no statistically significant difference in the SAH grades among different SAH groups (supplemental Fig. Ⅱ).

**References**

1.Xu W, Mo J, Ocak U, et al. Activation of Melanocortin 1 Receptor Attenuates Early Brain Injury in a Rat Model of Subarachnoid Hemorrhage viathe Suppression of Neuroinflammation through AMPK/TBK1/NF-κB Pathway in Rats. Neurotherapeutics. 2020; 17(1): 294‐308.

2.Peng J, Pang J, Huang L, et al. LRP1 activation attenuates white matter injury by modulating microglial polarization through Shc1/PI3K/Akt pathway after subarachnoid hemorrhage in rats. Redox Biol. 2019; 21: 101121.

3.Xu, W., et al., Sodium Benzoate Attenuates Secondary Brain Injury by Inhibiting Neuronal Apoptosis and Reducing Mitochondria-Mediated Oxidative Stress in a Rat Model of Intracerebral Hemorrhage: Possible Involvement of DJ-1/Akt/IKK/NFkappaB Pathway. Front Mol Neurosci, 2019. 12: p. 105.

4. Curzer, H.J., et al., The Three Rs of Animal Research: What they Mean for the Institutional Animal Care and Use Committee and Why. Sci Eng Ethics, 2016. 22(2): p. 549-65.
